# Bioorthogonal Suzuki–Miyaura Cross-linking: Transforming Responsive Hydrogels into Permanent Polymer Networks

**DOI:** 10.1021/acs.biomac.5c01053

**Published:** 2025-11-10

**Authors:** Anastasia Anagnostou, George Pasparakis

**Affiliations:** Department of Chemical Engineering, 37795 University of Patras, Caratheodory 1, University Campus, GR 265 04 Patras, Greece

## Abstract

In this study, we present a novel approach to convert reversible polymer networks into stable, nonresponsive networks using bio-orthogonal Suzuki–Miyaura coupling (SMC). By leveraging boronic acids, which form reversible boronate esters with cis-diols for shear-thinning injectable gels and serve as SMC substrates to create stable C–C bonds, we achieve switching from responsive to nonresponsive behavior. We demonstrate the concept with a responsive precursor based on sodium alginate modified with phenyl boronic acid, cross-linked with three model iodide-functionalized (macro-)­molecules that exhibit irreversible gelation under bio-orthogonal conditions. The resulting networks exhibit robust mechanical stability, minimal pH and temperature responsiveness, high degradation resistance and excellent hemocompatibility. The proposed approach underlines the potency of SMC as a robust synthetic strategy toward the synthesis and transformation of polymer networks for advanced biomedical applications.

## Graphical abstract

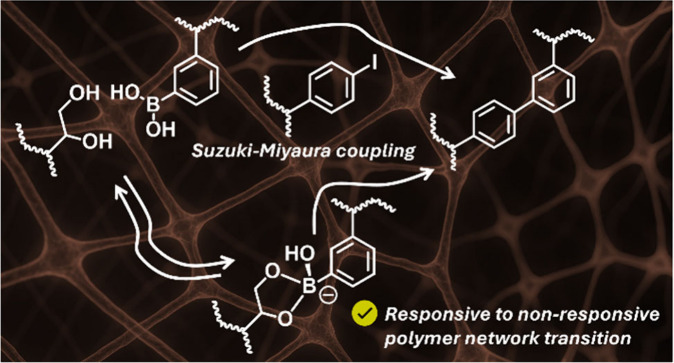

## 1 Introduction

Hydrogels constitute a ubiquitous class of polymer networks that find a multitude of applications in the biomedical field owing to their physicochemical properties that can be tailored to resemble physiological tissue and their facile synthesis through a plethora of polymer chain cross-linking strategies.^1,2^ The latter can be largely classified to covalent and noncovalent means to form responsive polymer networks with adaptable properties based on stimuli cues (i.e., temperature, pH, ionic gradients, etc.) or nonresponsive networks of permanently “fixed” behavior irrespective of the exposed conditions.^3,4^ Practical synthetic strategies to access switching from responsive to nonresponsive behavioral patterns has not been explored despite the obvious advantages in terms or functional versatility and complexity.^5^ For example, a hydrogel that exhibits transient shear thinning to enable injectability but can be fixated in a permanent state on demand could have obvious benefits in a diverse set of applications i.e. in injectable biomaterials, smart bioadhesives, as well as smart inks for additive manufacturing. Here, we report on a versatile strategy to transform reversible polymer networks to permanent nonresponsive networks that can withstand changes of exposure conditions. Our strategy comprises the exploitation of boronic acids that can form reversible boronate esters with cis-diol residues and can also act as efficient Suzuki-Miyaura coupling (SMC) substrates to enable the formation of stable C–C bonds (Scheme 1). Although the boronic acid – diol motif has been explored as a reversible cross-linking mechanism to form weak albeit reversible gels with shear-thinning properties enabling injectability,^6–8^ the use of SMC to form polymer networks has not been previously explored.

**Scheme 1:**
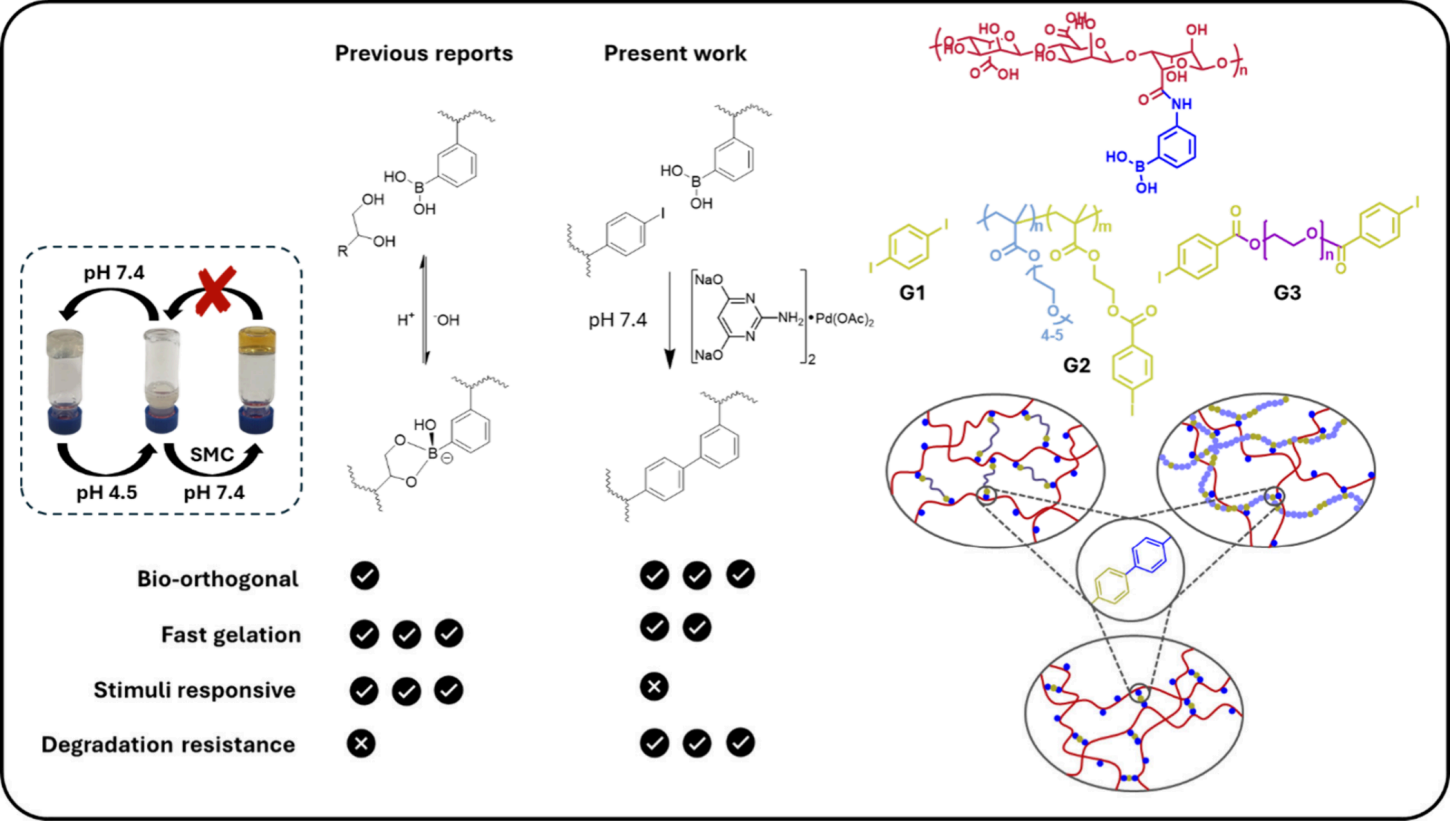
Proposed Concept of Transforming Responsive Boronate-Diol Polymer Networks to Permanently “Fixed” Stable Networks under Bio-orthogonal SMC Conditions

Boronic acids with an sp^2^-hybridized, trigonal planar boron, form cyclic boronate esters with cis-diols through a reversible condensation reaction where the diol’s hydroxyl groups nucleophilically attack the electron-deficient boron, transitioning it to an sp^3^-hybridized, tetrahedral intermediate, displacing water to create a five- or six-membered ring.^9,10^ Boronate-diol cross-linking is an attractive system as it exhibits fast gelation kinetics, it can withstand—to some extent^11^—the presence of free diols in biological media and is stimuli responsive in that the created bond is reversible in a pH-dependent manner (i.e., boronate esters are favored close or above the p*K*_a_ boronic acid, ca. 8.8) (Scheme 1).^12^ This is also the reason that boronate esters are prone to biodegradation in biological media, which is often a desired property, i.e. for controlled release applications.

In SMC, boronic acid undergoes base-activated transmetalation to transfer its carbon-based substituent (i.e., R from R-B­(OH)_2_) to a palladium catalyst after oxidative addition of an organohalide, followed by reductive elimination to form the coupled product and catalyst regeneration.^13–15^ Therefore, boronic acids could act with dual functionality both as a boronate ester building blocks and as SM cross-linking coupling moieties to switch from responsive to nonresponsive gelators in a fully biorthogonal manner.^16,17^ To this end, we demonstrate the concept with three model gelator systems (SM coupling networks, SCNs, Table S1), all based on a pH-responsive gelator^18^ of sodium alginate polymer chain modified with phenyl boronic acid groups (AlgBA, Table S2) cross-linked either with (i) a small commercially available bifunctional biphenyl iodide (G1), (ii) a random copolymer of poly­[(ethylene glycol) methacrylate-co-(4-iodobenzoyl) ethyl) methacrylate] synthesized in-house by free radical polymerization (G2, Table S3), or (iii) a bifunctional poly­(ethylene glycol) with iodobenzoyl end groups, also in-house synthesized (G3, Table S3) (Scheme 1, and Figures S2–S6 for synthesis and characterization). All three systems share the same Pd catalyst system comprising the sodium salt of 2-amino-4,6-dihydroxypyrimidine^19^ that yields acceptable conjugation efficiencies in aqueous media under mild conditions.

## 2 Materials and Methods

### 2.1 Materials

3-Aminophenylboronic acid hydrochloride (3-APBA.HCl, Aldrich), N-Hydroxysuccinimide (NHS, Fluorochem), 1-Ethyl-3-(3-(dimethylamino) propyl) carbodiimide (EDC, Alfa Aesar), 2- Morpholinoethanesulfonic acid (MES, Fluorochem), Sodium alginate (NaAlg, Aldrich), 2-hydroxyethyl methacrylate >99% (HEMA, Sigma-Aldrich), Triethylamine (TEA, Penta), 1,4-Diiodobenzene 98% (G1, Thermo Scientific), Palladium (ΙΙ) acetate 99.9% (Pd­(OAc)_2_, Thermo Scientific), 2-amino-4,6-dihydroxypyrimidine 98% (ADHP, Thermo Scientific), 4-iodobenzoyl chloride 98% (Thermo Scientific), Poly­(ethylene glycol) methyl ether methacrylate (MW 300, Sigma-Aldrich), Polyethylene glycol (MW 10000, Sigma-Aldrich), Potassium dihydrogen phosphate (KH_2_PO_4_, MERCK), Disodium hydrogen phosphate anhydrous (Na_2_HPO_4_, MERCK), Magnesium sulfate (MgSO_4_, Sigma-Aldrich), Dichloromethane (DCM, Fisher Scientific), Tetrahydrofuran (THF, Fischer Scientific), Calcium Chloride (CaCl_2_, MERCK), Sodium alginate (NaAlg, Aldrich) with a molecular weight range of 120,000–190,000 g/mol and a mannuronic/guluronic ratio (M/G) of 1.53 was dissolved at 7 *w*/*v*% in deionized water and was further purified against dialysis membrane (MWCO 12,000–14,000 Da) before being freeze-dried.

### 2.2 Catalyst Preparation

The palladium catalyst was prepared according to a previously reported protocol.^1^ 2-amino-4,6-dihydroxypyrimidine (13 mg, 0.1 mmol) was dissolved in NaOH solution (0.1 M, 2 mL) at 65 °C. Palladium acetate (11 mg, 0.05 mmol) was then added and the solution was stirred for 30 min at 65 °C. The orange solution was then allowed to cool to room temperature and was diluted to 5 mL with distilled water to give a stock 0.01 M catalyst solution. The stock catalyst (Pd­(OAc)_2_(ADHP)_2_) solution was stored at 4 °C.

### 2.3 Alginate-graft-Phenylboronic acid

3-Aminophenyl boronic acid (BA) conjugation on the alginate backbone was carried out by EDC/NHS carbodiimide coupling.^2^ Sodium alginate (746 mg, 0.005 mmol) was dissolved in 25 mL MES buffer solution (0.1M) and the pH was adjusted to 5.5 using HCl solution (0.1N). Following, EDC (521.5 mg, 2.720 mmol) and NHS (74.5 mg, 0.647 mmol) were added to the solution. Finally, 3-APBA was added to the mixture and the mixture was left stirring for 12h at rt. The resulting reaction solution was dialyzed (MWCO: 3500 Da), while the pH of the solution was maintained at 5.5. AlgBA was received through lyophilization with a yield of 95.5%. The product was characterized by using ^1^H NMR spectroscopy.

### 2.4 4-(4′-Iodobenzoyl)­ethyl Methacrylate (4-IEMA)

2-Hydroxyethyl methacrylate (HEMA) (0.204 g, 1.57 mmol), anhydrous triethylamine (0.364 mL, 2.62 mmol) in 4 mL anhydrous DCM were added to a 10 mL round-bottomed flask. The reaction mixture was degassed under nitrogen flow for 15 min. The reaction mixture was degassed under a nitrogen atmosphere for 15 min. Separately, 4-iodobenzoyl chloride (0.349 g,1.31 mmol)­was dissolved in 3 mL of anhydrous DCM. The HEMA solution was then cooled to −5 °C with stirring, and the 4-iodobenzoyl chloride solution was added dropwise. When the addition was completed, the ice bath was removed, and the reaction was left under stirring for 2 h at room temperature. Subsequently, the reaction mixture was again cooled to −5 °C and ∼5 mL of deionized water was added. The organic phase was washed with 0.1 M NaHCO_3_ (X1) and brine (X1), dried over MgSO_4_, filtered and concentrated. The crude product was passed through silica gel column using hexane and ethyl acetate (9:1 v/v) for further purification (hexane/ethyl acetate, 1:9, R_f_ 0.24) and was received with a yield of 55.5%. The product was characterized using ^1^H NMR and ^13^C NMR spectroscopy and was stored at −20 °C.

### 2.5 OEGMA300-r-IEMA (G2)

The copolymer was synthesized by free radical polymerization. In a 50 mL single-neck round-bottom flask, OEGMA_300_ (1.2 g, 4.016 mmol), 4-IEMA (160.6 mg, 0.446 mmol) and AIBN (5 mg, 0.03 mmol) were dissolved in THF (26 mL). The flask was sealed with a rubber septum and then purged with argon for ∼15 min. The flask was heated at 65 °C for 20 h under magnetic stirring. The reaction was stopped by exposing the solution in open air and the product was precipitated in cold hexane (250 mL). The final product (2) was obtained as a sticky colorless glue with a yield of 79.8% (1.086 g) and was characterized using ^1^H NMR spectroscopy and GPC.

### 2.6 PEG10000-I (G3)

The PEG macromer was functionalized with iodobenzoyl groups by the reaction of 4-iodobenzoyl chloride with the hydroxyl end groups of PEG. Polyethylene glycol (MW 10000, 5 g, 0.5 mmol) and triethylamine (0.3 mL, 2.1 mmol) were dissolved in 20 mL anhydrous DCM in a 25 mL round-bottom flask. The mixture was degassed under nitrogen flow for ∼15 min. After immersing the flask in an ice bath, 4-iodobenzoyl chloride (0.503 g, 2 mmol) dissolved in 10 mL of anhydrous DCM was added dropwise to the reaction under continuous stirring. The final solution was left stirring at rt for 12 h. The salt formed was removed through filtration. Then, the product was precipitated in cold ethyl ether as a white solid and isolated through vacuum filtration. It was left to dry at 55 °C for ∼1 d. The reaction yield was 79.2%, the degree of substitution was 87.3% and the final polymer was characterized using ^1^H NMR spectroscopy and GPC.

### 2.7 Hydrogel Formation via Suzuki–Miyaura Cross Coupling

General Procedure: The cross-coupling reactions resulting in the formation of hydrogels were performed by dissolving AlgBA in Na_2_HPO_4_ (70 mM) aqueous solution and then adding the di-iodine-functionalized molecule (G1, G2 or G3) also dissolved in Na_2_HPO_4_ solution (Table S1). The pH of the solution was adjusted to 7.4. Stock palladium catalyst Pd­(OAc)_2_(ADHP)_2_ (2 mM) was then added, and the mixture was stirred at 37 °C for 8 h.

### 2.8 ^1^H NMR

^1^H NMR spectra of the products were received using a Bruker Avance III HD Prodigy Ascend TM spectrometer at room temperature in D_2_O or CDCl_3_ solvent. ^1^H NMR spectra were obtained at 600.13 MHz and ^13^C NMR spectra at 150.90 MHz. Data were processed with MestReNova software.

### 2.9 Gel Permeation Chromatography (GPC)

Chromatograms were run at room temperature using THF as eluent with a flow rate of 0.5 mL/min. The detection was conducted using UV absorbance (FASMA 500 UV/vis detector). A universal calibration curve was created with polyethylene glycol (PEG) standards and the results were analyzed with Clarity software.

### 2.10 Fourier Transform Infrared (FTIR) Spectrometry

Fourier transform infrared (FTIR) experiments with dry samples were carried out using an IRTracer-100 FTIR (Shimadzu, Tokyo, Japan) FTIR spectrometer equipped with the ATR accessory MIRacle Single Reflection (Madison, WI, USA). The spectra were acquired after 20 scans at a resolution of 4 cm^–1^ and in the spectral range between 550 and 4000 cm^–1^. All measurements were carried out at room temperature.

### 2.11 Rheological Studies

The rheological behavior of hydrogels was evaluated at 37 °C using a stress-controlled AR-2000ex (TA Instruments) rheometer equipped with a solvent trap to minimize sample drying due to water evaporation with a cone–plate geometry (diameter 20 mm, angle 3°, truncation 111 μm). The experiments were performed in the linear viscoelastic region (LVR), which was determined by strain sweep tests at a frequency of 1 Hz. Hydrogel samples were loaded on a Peltier plate system able to control the experimental temperature with high accuracy (±0.1 °C). Data were processed with TRIOS software.

### 2.12 Degradation Study

Degradation studies were conducted by monitoring the storage modulus over a period of 28 days in both physiological and acidic pH (phosphate buffer at pH 7.4 and 4.5). In parallel, the dry mass of the samples was monitored to elucidate possible material loss. Rheological measurements were conducted with frequency sweep ranging from 0.1 to 100 rad/s at a strain of 0.1% (linear viscoelastic region) for each sample. At least five data points were collected for the linear elastic region and averaged to obtain the gel’s shear storage modulus. After testing (day 28), the gels were frozen in liquid nitrogen and lyophilized for 24 h before measuring the dry mass in order to compare it with their initial mass.

### 2.13 Swelling Assay

The swelling behavior of the hydrogel was assessed gravimetrically by measuring the mass of the absorbed water over time. The resulting hydrogels were initially fully dried and their mass was measured; then, they were immersed in PBS (7.4 and 4.5) at 37 °C until they reached equilibrium. At fixed time intervals, the samples were fetched out, the water on their surface was gently removed with filter paper and then weighed. The swelling degree of the hydrogel samples was assessed by comparing the weights of the swollen sample (*m*_w_) and dried sample (*m*_d_) and calculated according to eq 1.

1 $$\mathrm{Swelling}\;\mathrm{Degree}=\frac{m_{w}}{m_{d}}$$

### 2.14 Viscometry

The polymer solutions (AlgBA) were prepared by dissolution of a known amount of polymer in a 0.1 M NaCl solution. Viscometry measurements of dilute solutions were performed on an Ubbelhode capillary viscosimeter (type 0c) that was immersed in a water bath previously equilibrated at 25 °C ± 0,1 °C using a Schott Gerate Viscometer (Type AVS 300). The AlgBA aqueous solutions used were in the range of 0 to 2.4 g/L.

### 2.15 Scanning Electron Microscopy (SEM) Analysis

All hydrogel samples were frozen in liquid nitrogen followed by freeze-drying. Fragments of the freeze-dried samples were attached to double-side self-adhesive carbon disc and mounted on a 25 mm aluminum stub. The samples were sputter-coated with gold and their morphology was observed using a scanning electron microscope (JEOL JSM-6610LV, SEM). The photos were used to determine the average pore size (through mean pore area measurement) and the % porosity (% area of pores) of the hydrogels by using ImageJ software. At least three surface photographs were used for each sample.

### 2.16 Hemolysis Assay

Hemolysis is the loss of membrane integrity of red blood cells (RBCs) leading to the leakage of hemoglobin (Hb) into blood plasma. Hemocompatibility is an essential property for biomedical materials. Five mL of blood from a healthy human donor were drawn directly into EDTA-coated Vacutainer tubes to prevent coagulation. For the hemolysis assay, the blood was centrifuged at 1000–1500 g for 10 min at 4 °C and washed three times with sterile PBS 1X (pH 7.4). SDS was used as positive control while PBS buffer was used as the negative control. The hydrogel samples were frozen in liquid nitrogen and freeze-dried for 12 h before exposure to blood. All the samples were incubated at 37 °C for 1 h with gentle shaking on a FALC shaker. After incubation, the tubes were centrifuged at 1500 rpm for 5 min to pellet the intact RBCs. The visual depiction of the tubes after centrifugation are presented in Figure 4b. The supernatant was then carefully collected and transferred to a new set of microcentrifuge tubes to measure the absorbance at 545 nm using the Varian Cary UV–vis spectrometer. The hemolysis rate (HR%) was calculated by eq eq 2. A mean hemolysis value of less than 5% was considered acceptable based on the ASTM F756 standard.

2 $$\mathrm{Hemolysis}\;\mathrm{Rate}\;\left(\%\right)=\frac{A_{S}-A_{N}}{A_{P}-A_{N}}\times 100$$

where *A*_S_ represents the absorbance value of the sample group, *A*_N_ represents the absorbance value of the negative control group, and *A*_P_ represents the absorbance value of the positive control group.

### 2.17 Statistical Analysis

The numerical data was statistically analyzed using one way ANOVA followed by Tukey’s multiple comparison test. A *p* value less than 0.05 was considered as statistically significant.

## 3 Results and Discussion

First, we established that the AlgBA precursor undergoes pH-dependent sol–gel transition at the same conditions required for SMC, to enable the transition from a pH-responsive gelator to a permanent nonresponsive hydrogel upon SMC (Figure S7). The transient gel formation of AlgBA and the SCNs just before the catalyst addition was also monitored by strain and frequency sweep experiments (Figure S8–S9). Then, we studied the gelation kinetics to confirm the SM-mediated formation of the SCNs under physiological conditions (pH 7.4, 37 °C, Figure 1a). SCN1 almost immediately reached gel state (within 17.6 min, gelation onset where *G*′ = *G*′′) presumably owing to the fast diffusion of the low molecular G1 cross-linker; SCN2 also showed relatively rapid gelation at 41.6 min while SCN3 reached gelation at 118.5 min. The gelation times are reasonably comparable with free radical polymerization systems that also form carbon–carbon bonds (see, for example, refs 20,21 ) but without truly biorthogonal mechanism given the high reactivity of the free radicals that are involved. From the maximum *G*′ values obtained we could determine a rough estimate of the cross-linking density (Mc) showing that the Suzuki coupling proceeds more efficiently with the order SCN2 > SCN3 > SCN1 (Table S4). Also, we observed good repeatability of the samples’ formation without significant variations in *G*′ values, showing adequate robustness of the coupling protocol.

**Figure 1:**
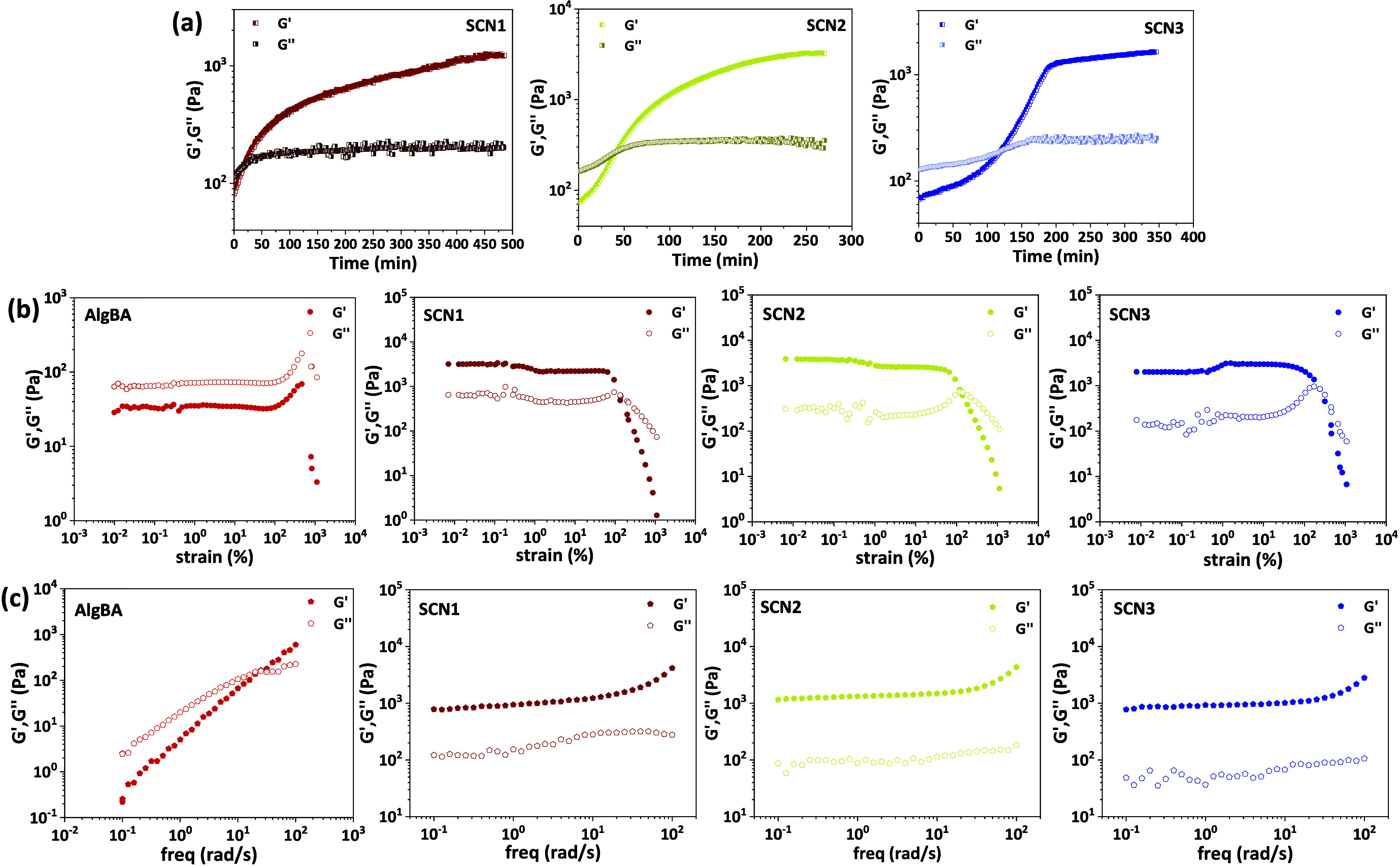
(a) Cross-linking kinetics of the SCNs, (b) Strain sweep experiments for AlgBA, and SCNs, and (c) Frequency sweep experiments for AlgBA, and SCNs.

The storage modulus plateaued at 1250 ± 15 Pa, 3260 ± 30 Pa and 1605 ± 35 Pa, for SCN1, SCN2, and SCN3, respectively. Interestingly, for the G3 gelator, the *G*′ reaches a plateau faster than the other two samples, even though the *G*′/*G*′′ crossover is somewhat delayed which may be attributed to the different number of anchoring points per chain (i.e., two iodide moieties per PEG chain for G3 vs, 3–5 moieties per chain for G2) which ultimately may affect the gelation kinetics. Also, it was possible to confirm the gelation conditions by monitoring the formation of the biphenyl bridge with ^1^H NMR and FT-IR (Figures S10–S11). Next, we determined the linear viscoelastic region (LVR) through strain sweep experiments. For the AlgBA sample the loss modulus (*G*′′) remains close but higher than the storage modulus (*G*′) during the LVR which suggests that the material exhibits sol-like behavior, as 37 °C is well above its gel–sol transition temperature (ca. 22 °C, Figure S12, also see below the effect of temperature).

For all SCNs, within the LVR, the elastic modulus dominates, indicating the materials retain their gel structure under small deformations; the wide LVR suggests strong internal network and robust cross-linking across all samples. Also, frequency sweep measurements were conducted to determine the hydrogels’ stability and strength. The AlgBA precursor shows frequency dependent storage modulus and a sol–gel transition as the frequency reaches 25.2 rad/s indicative of a transient network formation owing to the dynamic nature of the boronate-diol ester cross-linking (Figure 1c). Interestingly, the SCNs showed robust and stable gel-like behavior across the whole frequency range (0.1–1000 rad/s) indicative of the permanent C–C bond formation upon SMC (Figure 1c).

The halting of the temperature-dependent gel–sol transition as a result of SMC was also probed. In line with previous reports,^6,22^ it was confirmed that boronate esters tend to dissociate with temperature as evidenced by the tan­(δ) increase with temperature that induced gel–sol transition (tan­(δ) > 1) at ca. 22 °C (Figure 2a). In contrast, all SCNs retain their network integrity (tan­(δ) < 1) across the tested temperature range (Figure 2a and Figure S12). A slightly decreasing value of tan­(δ) as the temperature increases implies gel strengthening which may be attributed to increased chain mobility that assists the completion of the SM coupling within the network.

**Figure 2:**
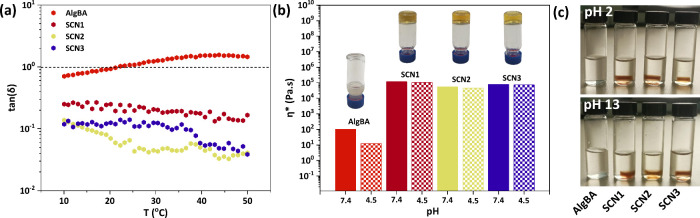
(a) tan­(δ) versus temperature of the samples (before and after hydrogel formation with G1, G2 and G3 (with a rate of 1 °C/min at pH 7.4), (b) complex viscosity as a function of pH, and (c) Images of AlgBA and SCNs at pH 2 and 13.

Next, we compared the effect of pH on the complex viscosity of the samples (η*). AlgBA showed a dramatic decrease of 88% at acidic pH due to the dissociation of the boronate ester bonds leading to sol-like behavior. On the other hand, SCN1, SCN2, and SCN3 experienced only 11, 16, 3% reduction of η*, respectively. Even with this decrease, the complex viscosity remained 4 orders of magnitude higher than that of the one of AlgBA (Figure 2b). Hence, we further challenged the samples by exposure at extreme pH conditions of 2 and 13, for 2 days. The SCNs appeared to remain structurally intact under both highly acidic and basic conditions, in stark contrast to the AlgBA sample that underwent complete dissolution even at pH 13 which is well above the p*K*_a_ of the phenylboronic acid residue (p*K*_a_ 8.8^9^) (Figure 2c).

We further evaluated the degradation resistance of the SCNs by monitoring the storage modulus for a 28-day period at pH 4.5 and 7.4. Specifically, for the SCN1 (using G1 as a cross-linker), the storage modulus decreased by 29.6% (990.4 ± 125 Pa) and by 30.3% (1001.6 ± 19 Pa) at pH 7.4 and pH 4.5, respectively (Figure 3a). For SCN2 the *G*′ value decreased by 12.3% (2190.4 ± 54 Pa) for pH 7.4 and by 32.1% (1680.1 ± 138 Pa) for pH 4.5; finally, the storage modulus of SCN3 was reduced by 23.5% (1110.8 ± 65 Pa) and 26.9% (1100.8 ± 98 Pa) at pH of 7.4 and 4.5, respectively (Figure 3a). It seems there is no clear effect of the pH and hence we attribute the decrease of the storage moduli to relaxation effects from the prolonged immersion times. However, it seems that hydrolysis effect may contribute to the reduction of the *G*′. We found that the Mz values of G2 and G3 (G1 was excluded as it is a low molar mass compound) remained almost unchanged for 28 days at pH 4.5 and 7.4 (Figure S13) whereas the intrinsic viscosity of the AlgBA component was significantly reduced (25% and 32%, at pH 7.7 and 4.5, respectively, Table S5). Therefore, possible hydrolysis events should be attributed to the alginate backbone and not to the more stable polymethacrylate and PEG chains. The absence of material loss under these conditions was evidenced by measuring the dry mass of the samples at days 0 and 28, which did not show significant differences (Figure 3b).

**Figure 3:**
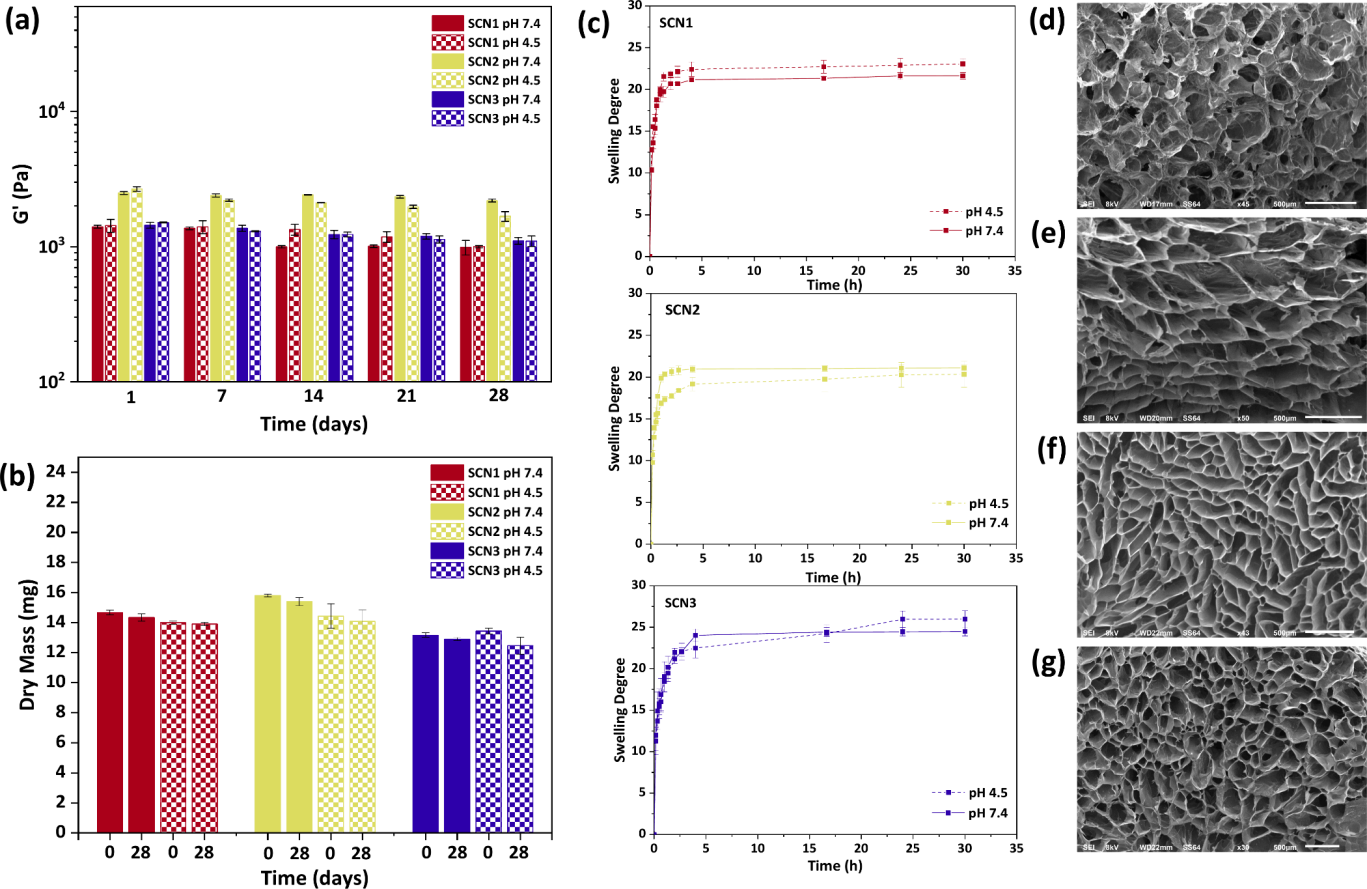
(a) Storage modulus (*G*′) of the final hydrogels over 28 days, (b) Measured dry mass of the initial hydrogels and the hydrogels after 28 days in pH 7.4 and 4.5, (c) Swelling behavior of each hydrogel at pH 7.4 and 4.5 (*n* = 3, *p* < 0.05), SEM images of AlgBA (d), SCN1 (e), SCN2 (f) and SCN3 (g) (bar scale: 500 μm).

Also, the SCNs showed only partial swelling in the first 2–4 h of immersion followed by a plateau irrespective of the pH implying the formation of stable networks with limited responsive effects of the residual pendant ionizable groups (i.e., free carboxyl groups, and phenyl boronic acids) that could otherwise impact the responsive properties of the networks (Figure 3c). Essentially, SMC results in the transformation of the polymer networks into stable permanently “fixed” irreversible structures.

The texture and morphology of the SCNs was studied by SEM which revealed a highly porous structure across all samples (Figure 3d-f). It is evident that the SCNs exhibit more distinctly defined pores than AlgBA. Following SM cross-linking, the D_pore_ value appears to be increased and tends to increase with the length of the gelator used. More precisely, for the AlgBA network the D_pore_ value was 51.4 ± 14 μm and for the SCN1, SCN2 and SCN3 was 94.6 ± 8 μm, 184.3 ± 14 μm and 224.9 ± 12 μm, respectively. The SCNs show similar porosity (%) values (50.4 ± 2% for SCN1, 56.2 ± 4% for SCN2, and 51.6 ± 11% for SCN3), slightly higher than AlgBA (41.8 ± 3%), though the differences are not statistically significant (Figure S14). We attribute these results to the augmented endurance of the SCNs to sublimation effects during lyophilization.

Finally, we performed an in vitro hemocompatibility assay to evaluate the blood compatibility of the SCNs. The results pointed out the concentration-dependent hemolysis induction which, however, remained below the acceptable 5% cytotoxicity threshold^23^ even at extremely high concentrations of 15 mg·mL^–1^ hemolysis did not exceed 1.5% (Figure 4a). Further, to underline the bio-orthogonality of the SMC process, we applied the gelation protocol in whole blood, showing the successful formation of SCNs with all three gelators (Figure 4c).

**Figure 4:**
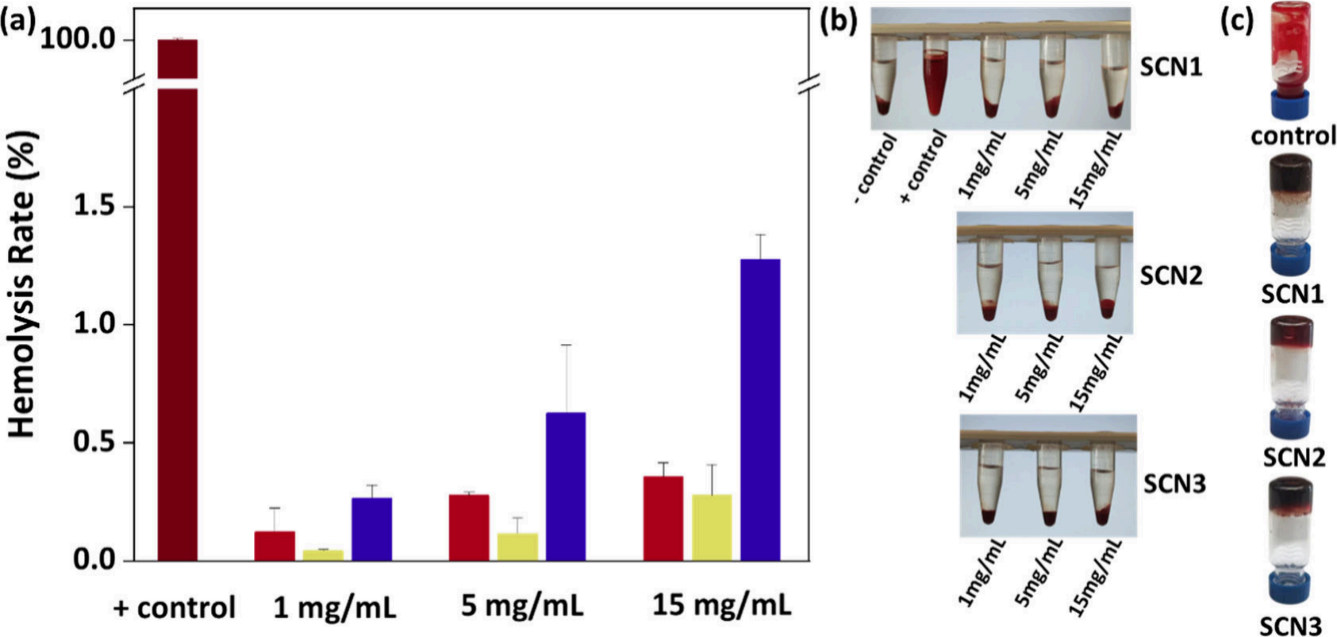
(a) Hemolysis rate as a function of hydrogel concentration (*n* = 3, *p* < 0.05), (b) Representative samples after hemolysis assay, and (c) SCNs formed in whole blood.

## 4 Conclusions

In conclusion, for the first time we report on the formation of polymer networks with SMC facilitating stable C–C cross-links. Starting from the dynamic boronate-diol system, it was possible to transform a responsive polymer network into a permanently irreversible hydrogel under mild and bio-orthogonal conditions. Potentially, our system can be combined with a variety of chemical synthons to produce hydrogels responsive to different stimuli (pH, light, temperature), depending on the selection of building blocks. We anticipate that our strategy will extend the paradigm of responsive materials beyond mere adaptation to stimuli, enabling transient-to-permanent state transitions. This adds an additional layer of complexity to smart materials, broadening their potential in advanced biomedical applications and we expect the approach to be widely adopted owing to the simplicity and robustness that SM reactions exert.

## Supplementary Material



## Notes

^1^H and ^13^C NMR, FTIR, GPC and additional rheological data (PDF)
