# Supplementary material for: Bioorthogonal Suzuki–Miyaura Cross-linking: Transforming Responsive Hydrogels into Permanent Polymer Networks
Source: Biomacromolecules. 2025 Nov 10;26(12):8389–95. doi: 10.1021/acs.biomac.5c01053 (PMC12690507; doi:10.1021/acs.biomac.5c01053)
Supplement: Supplementary file 1 [file bm5c01053_si_001.pdf]

SUPPORTING INFORMATION

Bioorthogonal Suzuki-Miyaura Crosslinking: Transforming Responsive Hydrogels into Permanent Polymer Networks

Anastasia Anagnostou and George Pasparakis*

Department of Chemical Engineering
University of Patras
Caratheodory 1, University Campus, GR 265 04 Patras, Greece
E-mail: gpasp@chemeng.upatras.gr

Experimental

Table S1. Crosslinking reaction stoichiometry

Hydrogel	AlgBA (w/v%)	Gelator	AlgBA (equiv.)	Gelator (equiv.)	DMSO (v/v%)*
**SCN1**	3	G1	1	10	1.4
**SCN2**	3	G2	1	10	-
**SCN3**	3	G3	1	10	-

* DMSO was used to solubilize and disperse 1,4-diiodobenzene in the aqueous mixture

Characterization

The molar composition (% mol/mol) of the grafting BA units onto the NaAlg was determined using ^{1}H NMR, by integrating the characteristic areas of the four protons of the phenyl ring of BA and the four protons of the NaAlg ring. Weight composition (% w/w) of the graft polymer was calculated by the molar composition by utilizing the Mw of the building units of sodium alginate and the Mw of the BA moieties.

The molecular characteristics of the final BA grafted Sodium Alginate are presented in Table S2, and the molecular weight of the material was calculated by equation eq. 3 as follows:

$$M_{w,cop} = \frac{M_{w,NaAlg}}{\%wt\ NaAlg} \quad \text{(eq. 3)}$$

where $M_{w,NaAlg}$= 140,000 kDa.

Table S2. Molecular Characteristics of AlgBA

Polymer	M_w (g/mol)^a	%Weight Composition NaAlg/BA (wt/wt)	%mol Composition NaAlg/BA (mol/mol)
NaAlg-g-BA	185,700	75.4/24.6	72.9/27.1

^{a} *Calculated by equation (eq. 3)*

The molecular characteristics of the synthesized gelators (G2 & G3) retrieved by GPC are presented in Table S3:

Table S3. Molecular Characteristics of G2 and G3 Polymers

Polymer	Mn (Da)	Đ
**OEGMA300-r-IEMA (G2)**	11580	1.40
**PEG10000-I (G3)**	11000	1.20

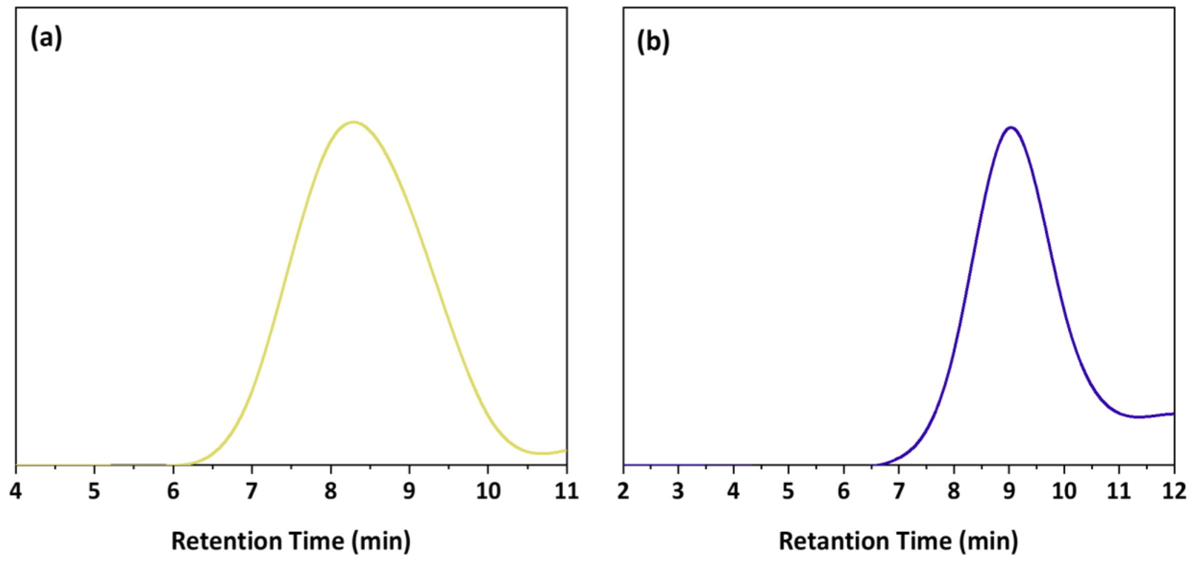

Figure S1. GPC chromatographs of (a) G2 and (b) G3 gelators.

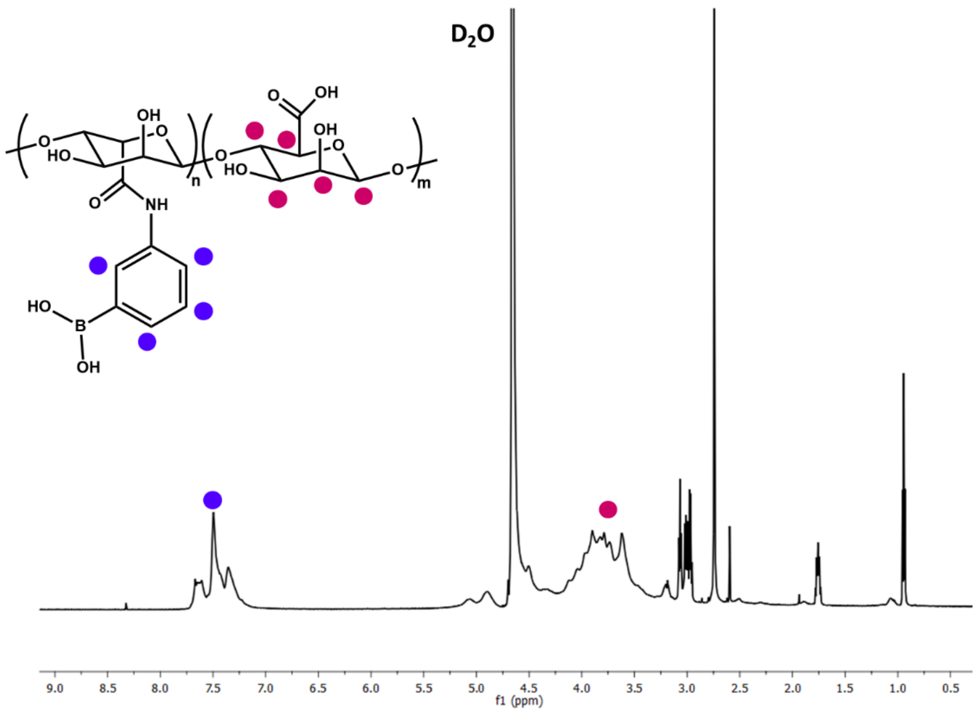

Figure S2. ^{1}H-NMR spectrum of sodium alginate-graft-phenylboronic acid (AlgBA).

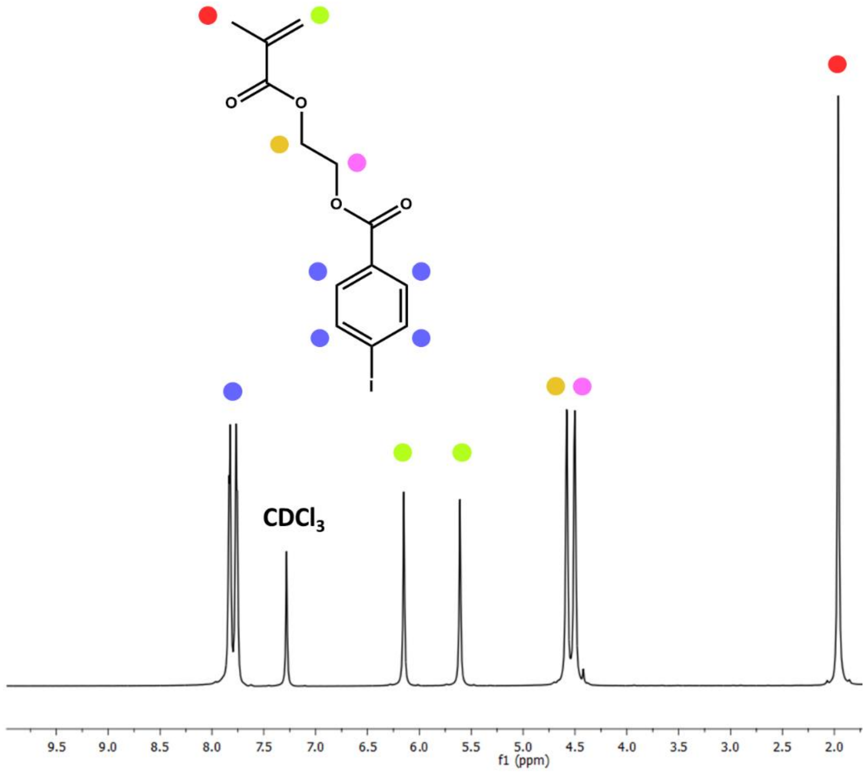

Figure S3. ^{1}H-NMR spectrum of 4-(4'-iodobenzoyl)ethyl methacrylate (4-IEMA).

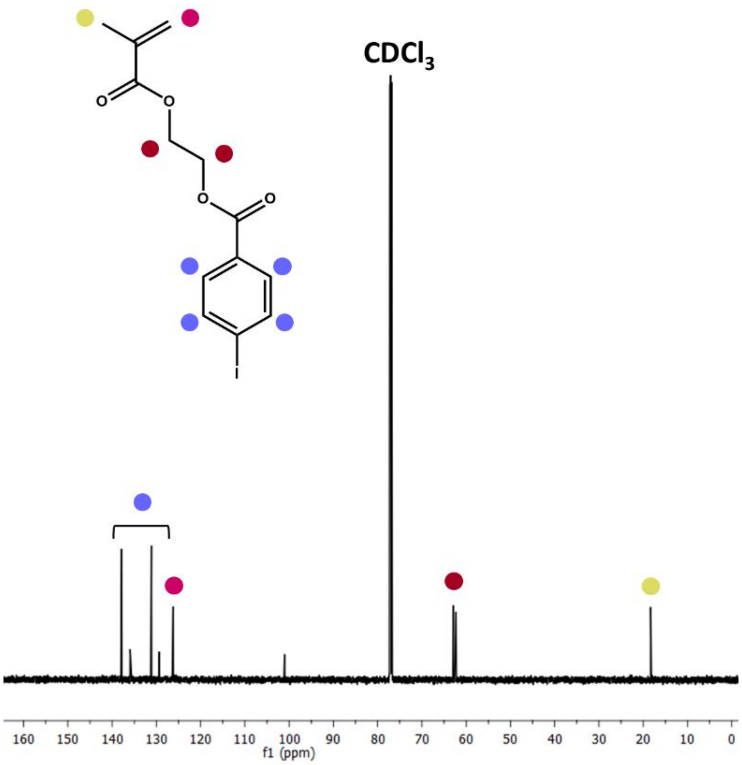

Figure S4. ^{13}C-NMR spectrum of 4-(4'-iodobenzoyl)ethyl methacrylate (4-IEMA).

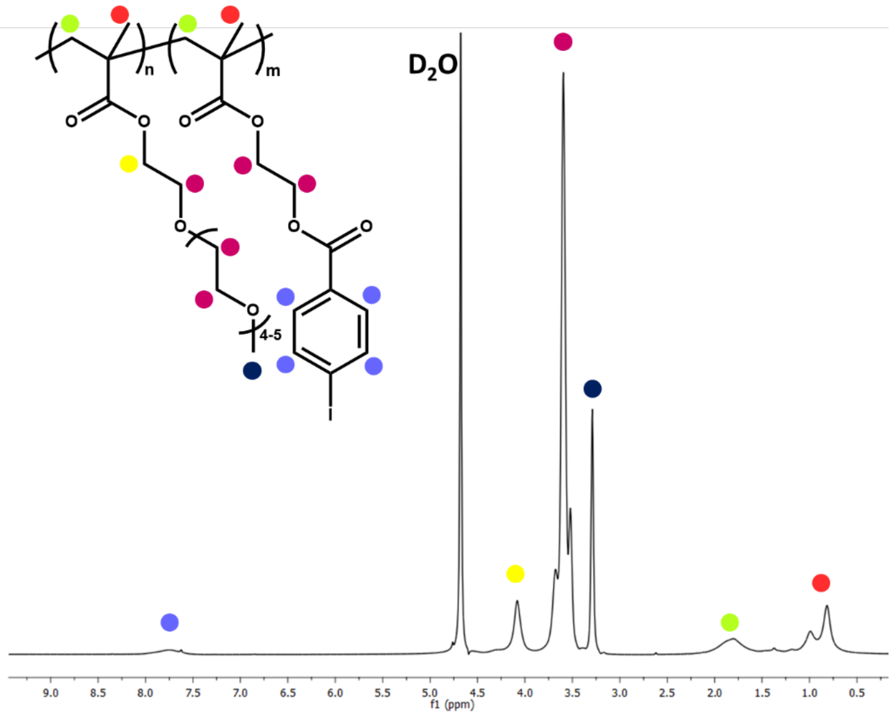

Figure S5. ^{1}H-NMR spectrum of $OEGMA_{300}$-r-IEMA (G2) (n=92%, m=8%).

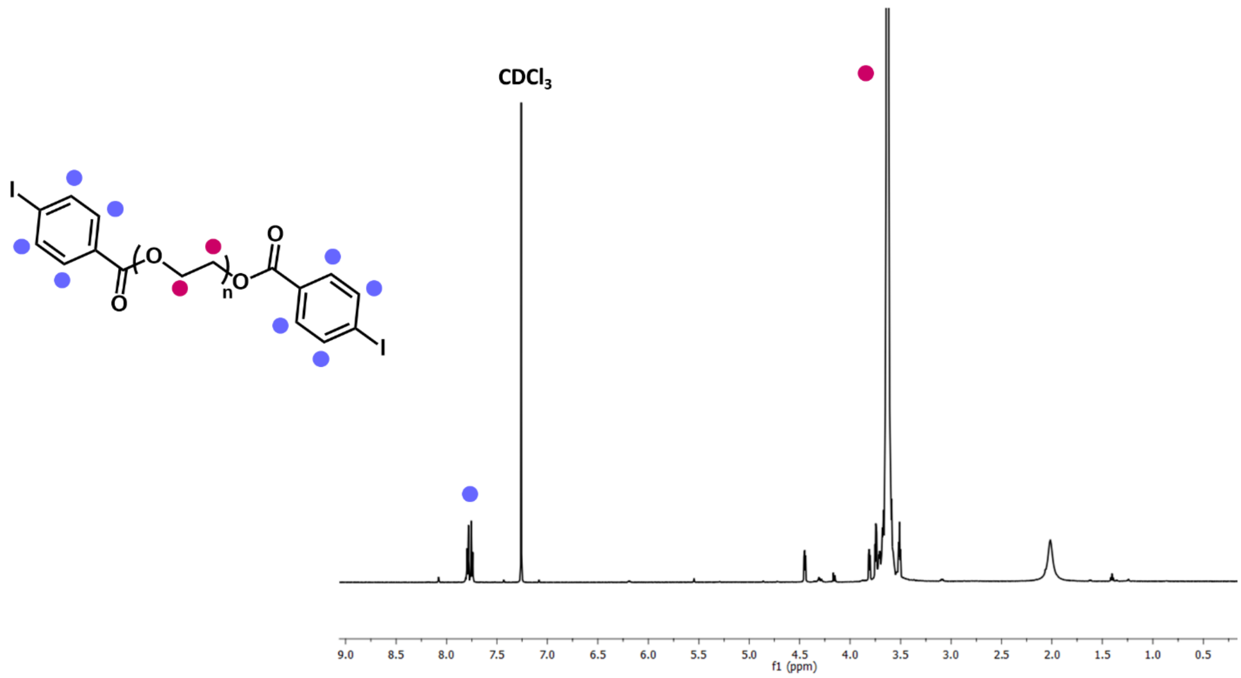

Figure S6. ^{1}H-NMR spectrum of PEG_{10000}-I (n~225).

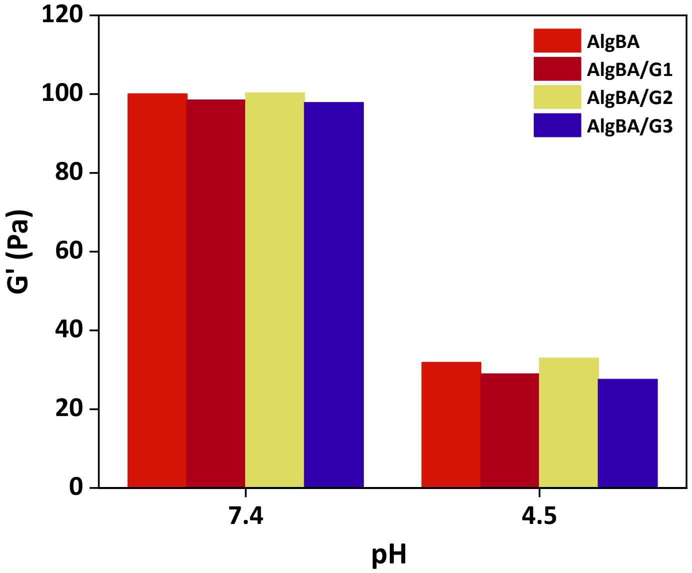

Figure S7. Storage modulus of AlgBA as a function of pH of (a) AlgBA and AlgBA mixed with each gelator used (G1, G2, and G3) before SMC catalyst addition showing their switchable gel-sol transition.

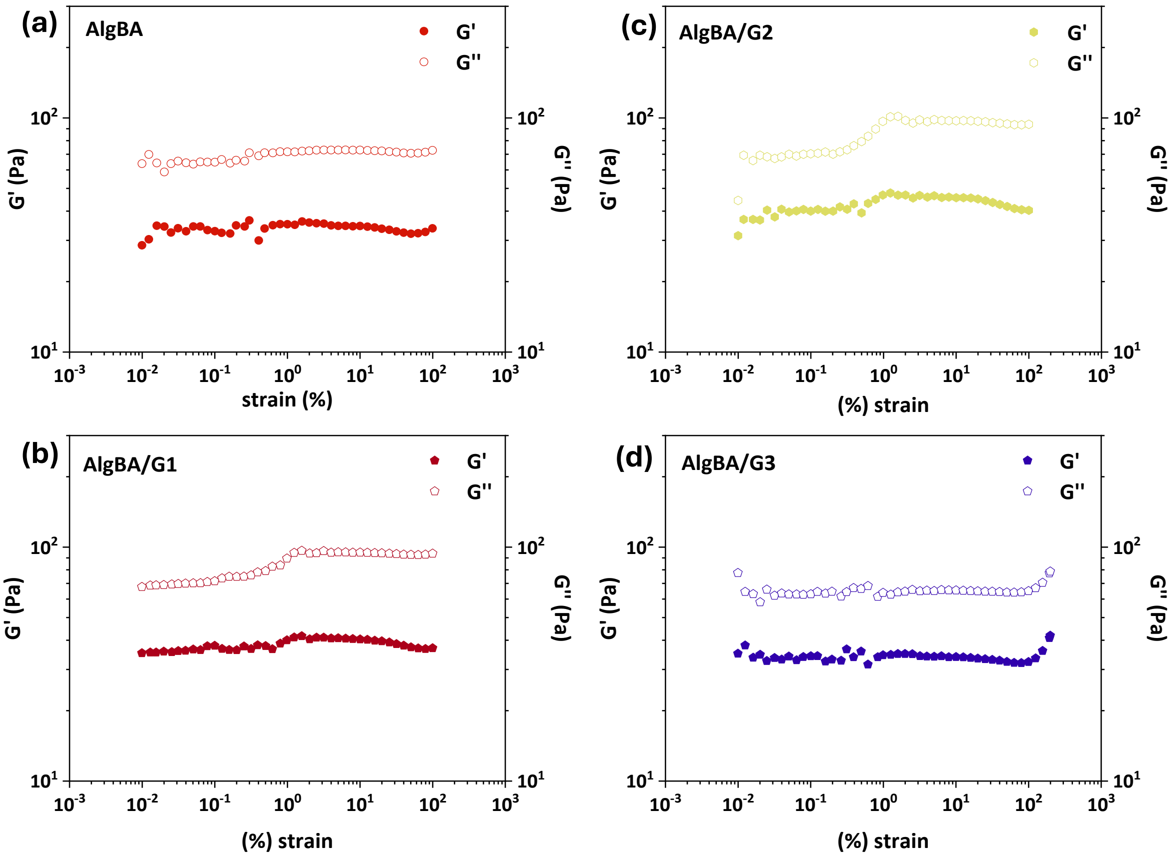

Figure S8. Storage and loss modulus as a function of % strain of (a) AlgBA and AlgBA mixed with each gelator used (b) G1, (c) G2 and (d) G3 before catalyst addition.

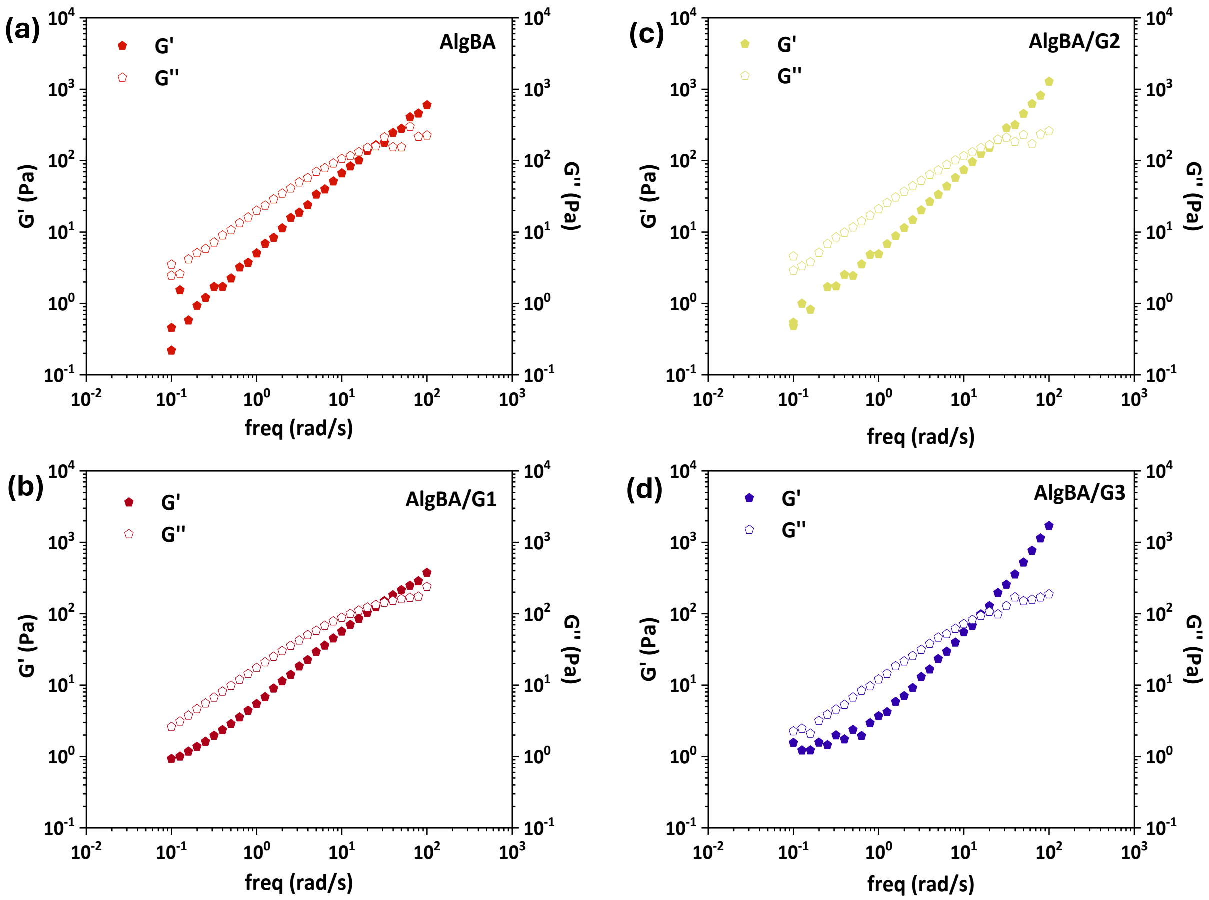

Figure S9. Storage and loss modulus as a function of angular frequency (rad/s) of (a) AlgBA and AlgBA mixed with each gelator used (b) G1, (c) G2 and (d) G3 before catalyst addition (0.1% strain).

To verify the SMC, the cross-coupling protocol was performed in situ in NMR tubes using 3-aminophenylboronic acid hydrochloride (3-APBA.HCl) in place of AlgBA combined with G2; this reaction mixture provided the clearest reading of the biphenyl product after SCN completion (Figure S9).

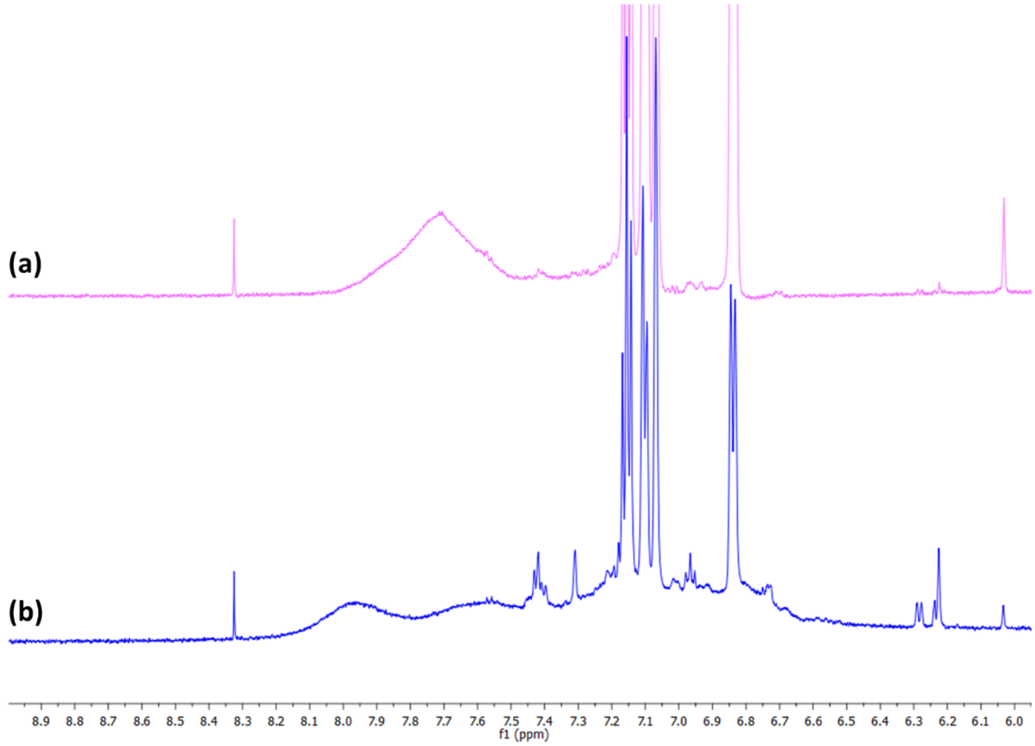

Figure S10. ^{1}H-NMR in D_2O before (a) and after 10 hours of (b) SM coupling reaction between G2 and 3-APBA.HCl showing biphenyl bridge at ca. 8.0 ppm as broad signal.[3]

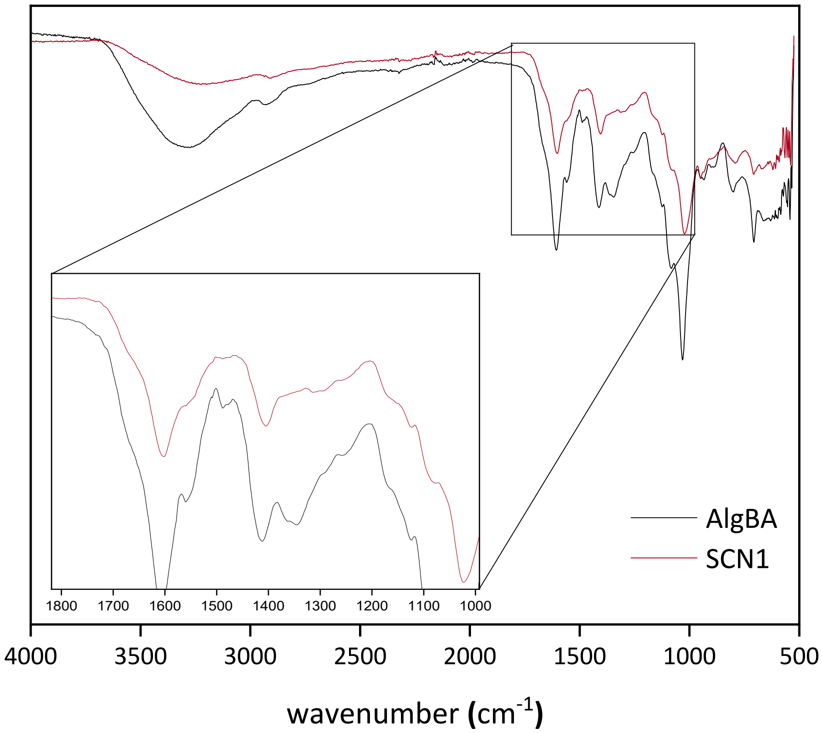

Figure S11. FTIR spectra of AlgBA and SCN1. Disappearance of the 1350 cm-1 peak of the B-O stretching after SMC for the SCN1 sample. For samples SCN2 and SCN3, the B-O stretching signal at ~1350cm-1 overlaps with the CH_2 wagging/scissoring vibration from the ethylene oxide repeating units that can be found in both G2 and G3 structures and hence FTIR was not conclusive for these samples.

Crosslinking density

To determine the molecular weight between crosslinks (or crosslinking density) we use the following formula:

$$Mc = \frac{c_p RT}{G'} \qquad \text{(eq. 4)}$$

where Mc is the molecular weight between crosslinks (or crosslinking density), Cp is the polymer, R is the gas constant (8.314 m^3 Pa mol^{-1} K^{-1}) and T is the temperature (K) and as G' we consider the maximum plateaued values. Based on this formula we obtain a rough estimate of the Mc for each sample:

Table S4. Estimation of the molecular weight between crosslinks.

Sample	Mc (g/mol)
SCN1	61,900
SCN2	23,700
SCN3	48,200

Based on these results we can conclude that the Suzuki-coupling reaction is more efficient with the order SCN2>SCN3>SCN1.

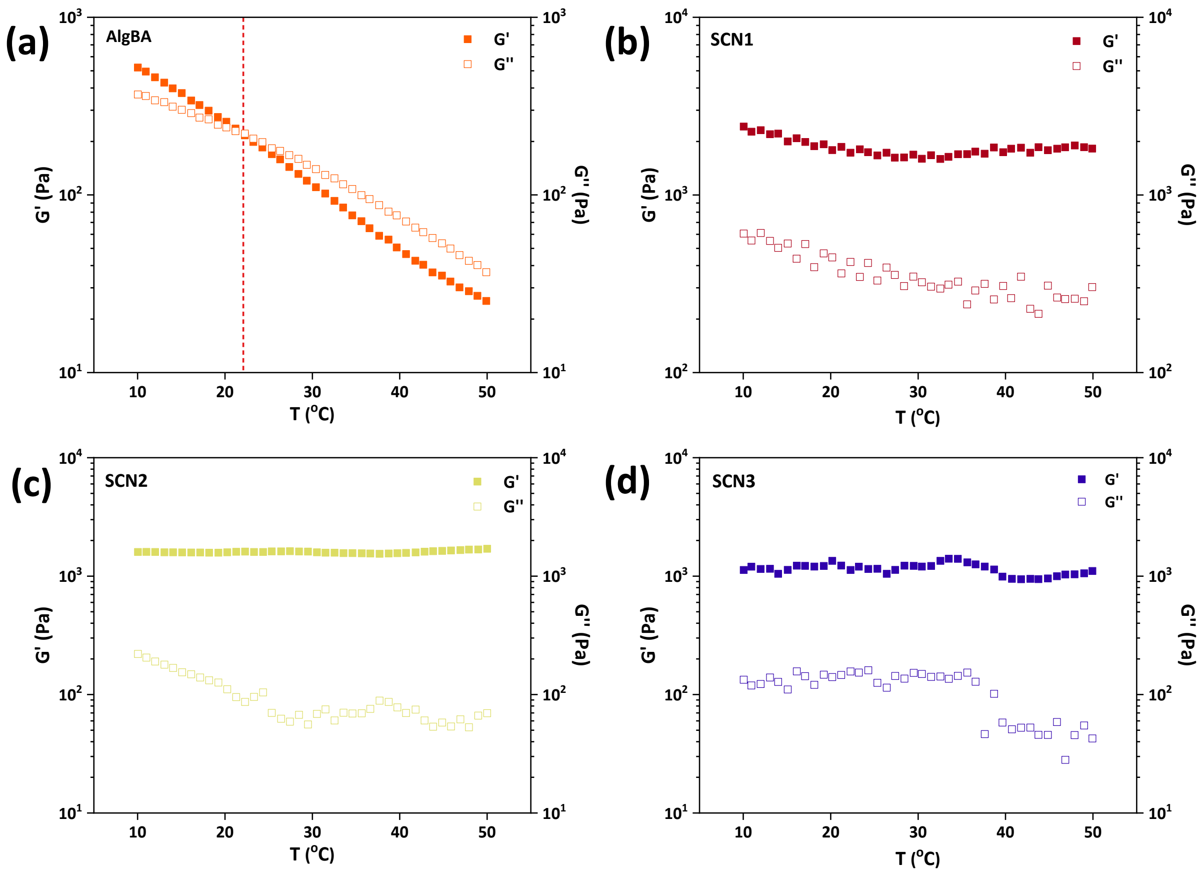

Figure S12. Storage and loss modulus behavior during temperature sweep of AlgBA and SCNs.

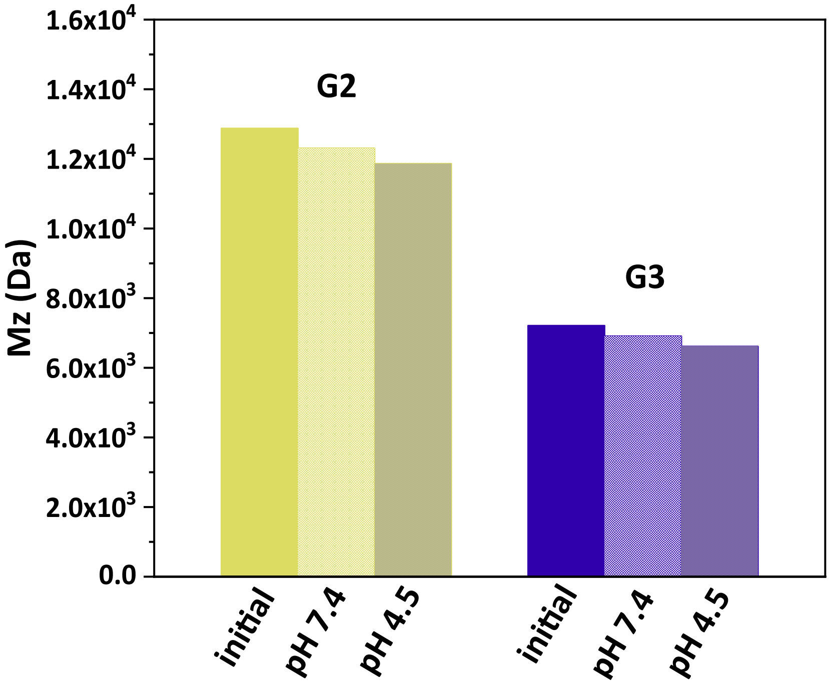

Figure S13. Mz values of G2 and G3 initially and after immersion in PBS (pH 7.4 & 4.5) for 28 days.

Table S5. Intrinsic viscosity % change of AlgBA at different immersion conditions.

**Sample**	**[η] (ml/g)**	**% change**
Initial AlgBA	191	-
AlgBA in pH 7.4	129	32
AlgBA in pH 4.5	143	25

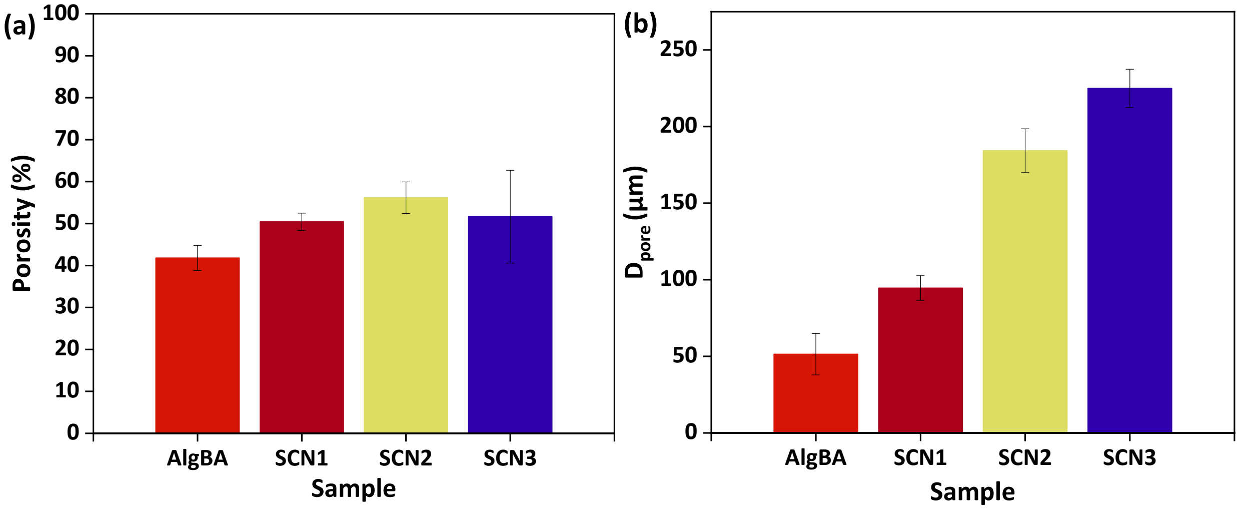

Figure S134. (a) % Porosity of the tested hydrogel samples ($p>0.05$) and (b) Average pore sizes ($p<0.05$).

References

[1] J. M. Chalker, C. S. C. Wood, and B. G. Davis, "A Convenient Catalyst for Aqueous and Protein Suzuki−Miyaura Cross-Coupling," *J Am Chem Soc*, vol. 131, no. 45, pp. 16346–16347, Nov. 2009, doi: 10.1021/ja907150m.

[2] S. H. Hong *et al.*, "Dynamic Bonds between Boronic Acid and Alginate: Hydrogels with Stretchable, Self-Healing, Stimuli-Responsive, Remoldable, and Adhesive Properties," *Biomacromolecules*, vol. 19, no. 6, pp. 2053–2061, Jun. 2018, doi: 10.1021/acs.biomac.8b00144.

[3] C. C. Piras, P. Slavik, and D. K. Smith, "Self-Assembling Supramolecular Hybrid Hydrogel Beads," *Angewandte Chemie International Edition*, vol. 59, no. 2, pp. 853–859, Jan. 2020, doi: 10.1002/anie.201911404.
